# Reduced incretin effect precedes diabetes development following duodenopancreatectomy in individuals without diabetes

**DOI:** 10.1172/JCI175133

**Published:** 2024-03-12

**Authors:** Gianfranco Di Giuseppe, Laura Soldovieri, Gea Ciccarelli, Pietro Manuel Ferraro, Giuseppe Quero, Francesca Cinti, Umberto Capece, Simona Moffa, Enrico Celestino Nista, Antonio Gasbarrini, Andrea Mari, Sergio Alfieri, Vincenzo Tondolo, Alfredo Pontecorvi, Jens Juul Holst, Andrea Giaccari, Teresa Mezza

**Affiliations:** 1 Centre for Endocrine and Metabolic Diseases, Fondazione Policlinico Universitario Agostino Gemelli IRCCS, Rome, Italy.; 2 Dipartimento di Medicina e Chirurgia Traslazionale, Università Cattolica del Sacro Cuore, Rome, Italy.; 3 Sezione di Nefrologia – Università di Verona, Verona, Italy.; 4 Digestive Surgery Unit and; 5 Pancreas Unit, CEMAD, Fondazione Policlinico Universitario Agostino Gemelli IRCCS, Rome, Italy.; 6 Institute of Neuroscience, National Research Council of Italy, Padua, Italy.; 7 Digestive Surgery Unit, Ospedale Isola Tiberina — Gemelli Isola, Rome, Italy.; 8 Novo Nordisk Foundation Centre for Basic Metabolic Research, University of Copenhagen, Copenhagen, Denmark.

**Keywords:** Endocrinology, Metabolism, Beta cells, Diabetes, Glucose metabolism

## To the Editor:

The incretin effect (IE) is a key factor regulating β cell functional response and affecting the dynamics of insulin secretion (1). The main actors in the IE are the incretin hormones GIP and GLP-1, which are secreted by specialized enteroendocrine cells in response to glucose, amino acids, and lipids. It is well known that the IE is greatly reduced in type 2 diabetes (T2D), albeit with considerable variability (2). However, longitudinal studies investigating the long-term consequences of the impaired IE in individuals without diabetes or those who are prediabetic are still lacking. To identify possible latent impairments in the IE that could begin in the nondiabetic state, we conducted a study using acute surgical removal of β cell mass as a surrogate model of the β cell loss occurring during the natural history of T2D. 35 individuals without diabetes scheduled for pancreatoduodenectomy underwent an in-depth metabolic evaluation before and after surgery (Supplemental Methods and Supplemental Figure 2; supplemental material available online with this article; https://doi.org/10.1172/jci.insight.175133DS1). Based on postsurgical OGTT-derived glucose tolerance, we classified the individuals as having normal glucose tolerance (post-NGT) (*n =* 10), impaired glucose tolerance (post-IGT) (*n =* 15), or diabetes mellitus (post-DM) after surgery (*n =* 10). Baseline characteristics of study participants are shown in Supplemental Table 1.

**Figure 1:**
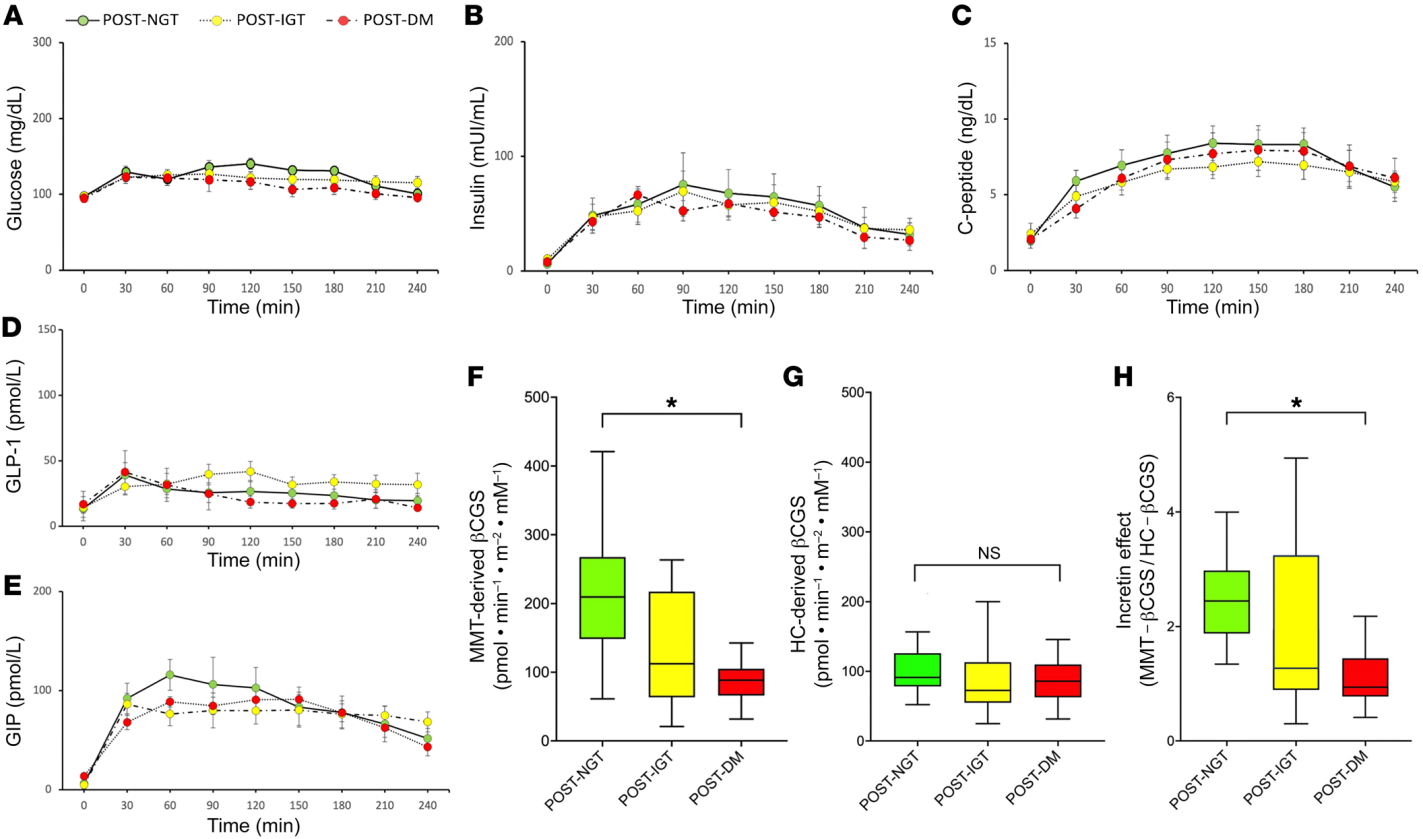
Functional defects predict diabetes onset after pancreatoduodenectomy despite normal hormone secretion. Glucose (**A**), insulin (**B**), C-peptide (**C**), GLP-1 (**D**), and GIP (**E**) levels during MMT in the post-NGT (solid lines, green circles), post-IGT (dotted lines, yellow circles), and post-DM (dotted-dashed lines, red circles) groups. MMT-derived βCGS (**F**), HC-derived βCGS (**G**), and IE (**H**) in the post-NGT (green), post-IGT (yellow), and post-DM (red) groups. *P < 0.05.

Before surgery, study participants had similar glucose, insulin, and C-peptide responses to a mixed-meal test (MMT) (Figure 1, A–C). Likewise, there were no differences in euglycemic hyperinsulinemic clamp–derived insulin sensitivity and in GLP-1 and GIP secretion over time among the 3 groups (Figure 1, D and E). Furthermore, we assessed the model-derived β cell glucose sensitivity (βCGS) during both intravenous and oral stimulation tests. There were no differences in hyperglycemic clamp–derived (HC-derived) βCGS among the 3 groups (Figure 1G); however, MMT-derived βCGS was significantly worse in patients who developed IGT and DM after surgery (Figure 1F, *P* < 0.01). We calculated the IE as the ratio of MMT-derived βCGS to HC-derived βCGS. The IE was significantly reduced in participants who developed IGT or diabetes after pancreatoduodenectomy (Figure 1H, *P* = 0.01). To verify whether the IE was dependent on functional β cell mass, we calculated HC-derived arginine-stimulated insulin secretion (ISR^ARG^) — an indirect index of functional β cell mass, expressing the ability of β cells to respond to a maximal stimulus — and regressed it against IE values. No correlations were found between the IE and ISR^ARG^ (Supplemental Figure 1), suggesting that impairments in the IE are not related to different β cell functional mass.

Our study demonstrates that preexisting defects in β cell response to incretins in individuals without diabetes predict the risk of developing impairments in glucose tolerance after acute β cell mass reduction by pancreatoduodenectomy. Specifically, we observed different metabolic trajectories based on differential β cellular responses to MMT and HC stimulation in a homogeneous cohort of individuals without diabetes. During both tests, we assessed βCGS, which measures the ability of β cells to cope with increased glucose levels by increasing insulin secretion. HC-derived βCGS was similar among the 3 groups, while there were significant differences in MMT-derived βCGS. Furthermore, only participants with a significant reduction in MMT-derived βCGS went on to develop IGT or overt hyperglycemia following acute reduction of β cell mass, confirming that the increase in insulin secretion depends not only on incretin levels, but also on β cell response to their action (3).

Moreover, our data showed comparable GIP and GLP-1 levels in response to MMT in the 3 groups, confirming no detectable major impairments in incretin secretion in this nondiabetic cohort but a significant variability in IE. In particular, only those participants with low IE developed IGT or DM. A reduced IE, therefore, seemed to predict the metabolic fate of these patients after surgery. These data suggest that the loss of the IE — unrelated to reduced circulating incretin levels — can also influence the natural history of T2D. Our findings support the hypothesis that a dysfunctional milieu, an “incretin resistance,” might be responsible (beyond incretin levels) for the impairment of the insulinotropic effect of incretins, as also observed in genome-wide association studies identifying possible genetic variants influencing islet sensitivity to incretins.

Caveats of this study are the extensibility of our metabolic model to the actual natural history of T2D and the use of study participants who are phenotypically different from most people developing β cell dysfunction in a context of obesity. In addition, the role of other incretin-like peptides cannot be excluded, even though this would not affect the measurement of the IE.

In conclusion, our data confirm that the IE varies greatly in humans without diabetes. Importantly, preexisting impairments in β cell function and the IE predict IGT and diabetes after partial pancreatectomy. This is consistent with the presence of “incretin resistance,” a dysfunctional milieu leading to the decreased sensitivity of islets to the action of incretin hormones. However, these early defects become clinically evident only when glucose homeostasis is affected by acute β cell loss, mimicking the pathological loss of functional β cell mass occurring over the natural history of T2D. We believe, therefore, that the present findings add important information to the knowledge on the pathogenesis of T2D. On the basis of our previous findings (1, 4), a direct link between a reduced IE and impaired first-phase insulin secretion cannot be excluded. Further studies will be necessary to confirm this hypothesis. Detecting early defects, even in people without diabetes, could help to identify individuals at higher risk of possible future glucose intolerance, and strategies to restore the incretin sensitivity of islets could be fundamental in preventing and treating diabetes according to a personalized medicine approach.

## Supplementary Material

Supplemental data

ICMJE disclosure forms

Supporting data values

## Version 1 03/12/2024

In-Press Preview

## Version 2 04/15/2024

Electronic publication
