# Supplementary material for: Reduced incretin effect precedes diabetes development following duodenopancreatectomy in individuals without diabetes
Source: J Clin Invest. 2024 Mar 12;134(8):e175133. doi: 10.1172/JCI175133 (PMC11014652; doi:10.1172/JCI175133)
Supplement: Supplemental data [file jci-134-175133-s229.pdf]

Supplemental Material

METHODS

Study population

Seventy-six subjects undergoing pylorus-preserving pancreatoduodenectomy were recruited from January 2019 to December 2022 at the Digestive Surgery Unit and studied at the Centre for Endocrine and Metabolic Diseases ("Agostino Gemelli" University Hospital, Rome, IT). Indications for surgery were periampullary tumors, pancreatic intraductal papillary tumors, mucinous cystic neoplasm of the pancreas, and nonfunctional pancreatic neuroendocrine tumors.

None of the patients enrolled had a known history of diabetes or was on any antidiabetic treatment. Only patients with normal cardiopulmonary and kidney function, as determined by medical history, physical examination, electrocardiography, estimated glomerular filtration rate and urinalysis, were included. Altered serum lipase and amylase levels prior to surgery, as well as morphologic criteria for pancreatitis at CT scan, were considered exclusion criteria. Patients with severe obesity (BMI > 40), uncontrolled hypertension and/or hypercholesterolemia were also excluded. Both males and females were included in this study, since sex was not considered as a biological variable. Patients underwent both an oral glucose tolerance test (OGTT) and HbA1c testing to exclude diabetes, according to the American Diabetes Association criteria. Eventually, as shown in Supplemental Figure 2, only 35 non-diabetic subjects (21 females; 14 males; mean age 59 ± 14 SEM) of the 76 enrolled were further investigated with an OGTT, a euglycemic hyperinsulinemic clamp (EHC), a mixed meal test (MMT) and a hyperglycemic clamp (HC) before surgery and an OGTT after surgery. Based on post-surgical OGTT-derived glucose tolerance, we could now classify the subjects as post-NGT (n = 10), post-IGT (n = 15), and post-DM (n = 10). Post-surgical OGTT evaluation was performed after an adequate recovery period (approximately 40 days after surgery), as determined by the normalization of inflammatory parameters, such as C-reactive protein, erythrocyte sedimentation rate, weight stability, absence of symptoms of abnormal intestinal motility or exocrine pancreatic deficiency. No differences were found among the 3 groups in the post-surgical histological diagnosis.

Oral Glucose Tolerance Test (OGTT)

A standard 75 g OGTT was performed with measurement of glucose, insulin and C-peptide at 0, 30, 60, 90, and 120 minutes after glucose load. All subjects underwent an OGTT both before and after surgery. As previously reported [16], to define the relationship between glucose metabolism after acute islet mass reduction and preexisting metabolic defects, we divided subjects according to their glucose tolerance after surgery, as determined by post-surgery OGTT. According to the American Diabetes Association classification, subjects whose 2-hour post-load glucose was below 140 mg/dl were defined as post-NGT, subjects whose 2-hour post-load glucose was 140–199 mg/dl were defined as post-IGT, and subjects whose 2-hour post load-glucose was equal to or higher than 200 mg/dl were defined as post-DM.

Mixed Meal Test (MMT)

A MMT was performed as previously described [32]. Patients were instructed to consume a liquid meal of 830 kcal (107 kcal from protein, 353 kcal from fat and 360 kcal from carbohydrates) within 15 mins. Blood samples were drawn twice in the fasting state and at 30-min intervals over the following 240 min (sample time 0, 30, 60, 90, 120, 150, 180, 210, and 240) for the measurement of plasma glucose, insulin, C-peptide, GIP and GLP-1.

During the MMT, β cell glucose sensitivity (MMT βCGS, $pmol \cdot min^{-1} m^{-2} \cdot mM^{-1}$) was assessed using a previously described mathematical model [34] In the MMT, βCGS is the slope of the relationship between insulin secretion and glucose concentration.

Euglycemic Hyperinsulinemic Clamp (EHC)

The EHC test was performed after a 12-hour overnight fast using 40 $mIU \cdot min^{-1} \cdot m^{-2}$ insulin of body surface, according to DeFronzo and colleagues [35]. A primed-constant infusion of insulin was administered (Actrapid HM, Novo Nordisk). The constant rate for the insulin infusion was reached within 10 minutes to achieve steady-state insulin levels; in the meantime, a variable infusion of 20% glucose was started via a separate infusion pump and the rate was adjusted, based on plasma glucose levels measured every 5 minutes, maintaining plasma glucose concentrations at fasting levels. During the last 20 minutes of the clamp procedure, plasma samples from blood drawn at 5- to 10-minute intervals were used to determine glucose and insulin concentrations.

During the EHC, whole-body peripheral glucose utilization was calculated during the last 30 minutes of the steady-state insulin infusion and was measured as the mean glucose infusion rate ($mg \cdot kg^{-1} \cdot min^{-1}$).

Hyperglycemic Clamp (HC)

The HC test was performed after a 12-hour overnight fast. Plasma glucose was clamped at a stable level of 125 mg/dl above fasting blood glucose concentration. The HC was started with a bolus dose of 200 mg/mL dextrose (150 mg/kg) administered into the antecubital vein. Blood was drawn from a cannulated dorsal hand vein on the opposite arm. Every 5 minutes, plasma glucose concentrations were measured and the infusion of 20% glucose was adjusted to achieve a stable glucose level of 125 mg/dl above the fasting value. Serum samples for insulin and C-peptide were drawn at 0, 2.5, 5, 7.5, 10, 15, 30, 60, 90, 120, 130, 140, and 150 minutes. At 120 minutes, a 5 g arginine bolus was administered to measure maximum C-peptide secretory capacity at a steady-state blood glucose concentration of 250 mg/dl. During the HC, insulin secretion was calculated from C-peptide deconvolution [16] β cell glucose sensitivity (HC βCGS, pmol·min^{-1}m^{-2}·mM^{-1}) was calculated as the ratio of the insulin secretion increment from the basal period to the 100-120 min period and the corresponding glucose concentration increment. Arginine-stimulated β cell secretory capacity (ISR^{ARG}, pmol/L), an indirect measurement of total functional β cell mass, was calculated as the C-peptide increment from 120 to 130 minutes during HC.

Biochemical measurements

At each sampling point, blood was collected in EDTA tubes for insulin and C-peptide measurement. Blood samples for GLP-1 were collected in tubes containing EDTA and a dipeptidyl peptidase-4 (DPP-4) inhibitor (Millipore, St. Charles, MO) to prevent the enzymatic degradation of GLP-1 (7-36) and GLP-1 (7-37) and immediately separated in a refrigerated centrifuge (1000 rpm for 10 min at 4°C). Plasma samples were divided into aliquots and stored at −80°C until analysis. Plasma glucose concentrations were determined bedside using the glucose oxidase technique on a glucose analyzer (Beckman Instruments, Palo Alto, CA). Insulin levels were determined using a commercial RIA kit (Medical System; Immulite DPC, Los Angeles, CA). Plasma C-peptide was measured by Auto-DELFIA Fluoroimmunoassay (Wallac, Turku, Finland). Plasma concentrations of GIP and total GLP-1 were measured using GIP and GLP-1 enzyme-linked immunosorbent assay (GIP and total GLP-1 NL-ELISA, Mercodia, Uppsala, Sweden).

Incretin Effect assessment

βCGS was calculated during both MMT and HC using consistent methods and units. Thus, MMT- and HC-derived βCGS represent the slope of the insulin secretion-glucose concentration

relationship, obtained with oral and intravenous glucose administration, respectively. In the present study, the incretin effect was estimated as the MMT-βCGS/HC-βCGS ratio.

Surgical Procedures

Pancreatoduodenectomy was performed according to the pylorus-preserving technique by Traverso-Longmire. Briefly, the pancreatic head, the entire duodenum, the common bile duct, and the gallbladder were removed *en bloc*, leaving an intact and functioning pylorus at the gastric outlet. All adjacent lymph nodes were carefully removed. The continuity of the gastrointestinal tract was restored by an end-to-side pancreatojejunostomy. Further downstream, an end-to-side hepaticojejunostomy and an end-to-side pylorojejunostomy were performed. The volume of pancreas removed during the surgery was constant (~50%), as previously reported by Schrader et al. [36]

Statistics

Continuous variables were summarized as mean ± SEM and categorical variables as frequencies and percentages, unless otherwise indicated. Normality of distribution was assessed by generation of histograms and quantile-quantile plots. Since samples did not deviate significantly from normality, differences in means across groups at baseline were tested by *ANOVA* and two-tailed Student *t test*. The relationship between variables was derived with linear regression analysis. For measurement of glucose, insulin, C-peptide, GLP-1 and GIP levels, we performed a second-level interaction analysis by including a product term of time × glucose tolerance in the model: we compared the effects of time using a linear mixed model for repeated measures, with each parameter as the dependent variable and time (analyzed as a categorical variable), and the product term of time × glucose tolerance to investigate interaction effects. A 2-tailed P value of less than 0.05 was considered statistically significant. Analyses were performed using Stata 15.1 (StataCorp).

**Subject characteristics**	**post-NGT (*n* = 10)**	**post-IGT (*n* = 15)**	**post-DM (*n* = 10)**	**P Value**
Mean age (y)	55.4 ± 5.61	62.2 ± 3.73	54.8 ± 4.30	-
Gender (F/M)	6/4	11/4	3/7	-
BMI (kg/m^2)	25.2 ± 1.23	27.3 ± 0.86	25.0 ± 1.10	0.17
Insulin sensitivity ($mg \cdot kg^{-1} \cdot min^{-1}$)	4.54 ± 0.66	5.15 ± 0.61	4.09 ± 0.51	0.44
Fasting glucose (mg/dl)	97 ± 3	97 ± 5	95 ± 3	0.91
Mean glucose (mg/dl)	121 ± 9	118 ± 7	109 ± 4	0.49
Fasting insulin (µUI/ml)	6.40 ± 1.11	10.1 ± 3.26	7.50 ± 1.61	0.57
Fasting C-peptide (ng/ml)	1.98 ± 0.25	2.36 ± 0.61	2.03 ± 0.26	0.82
HbA1c (mmol/mol)	37.5 ± 1.32	36.2 ± 1.62	34.8 ± 2.52	0.65
AUC GLP-1 ($pmol/l \cdot min^{-1}$)	6155 ± 1101	7947 ± 1076	5611 ± 1315	0.31
AUC GIP ($pmol/l \cdot min^{-1}$)	20184 ± 3294	17697 ± 2403	17728 ± 1489	0.74

Supplemental Table 1. Clinical and metabolic characteristics of non-diabetic subjects before surgery, classified according to glucose tolerance after surgery into post-NGT, post-IGT and post-DM. Data are represented as means ± SEM.

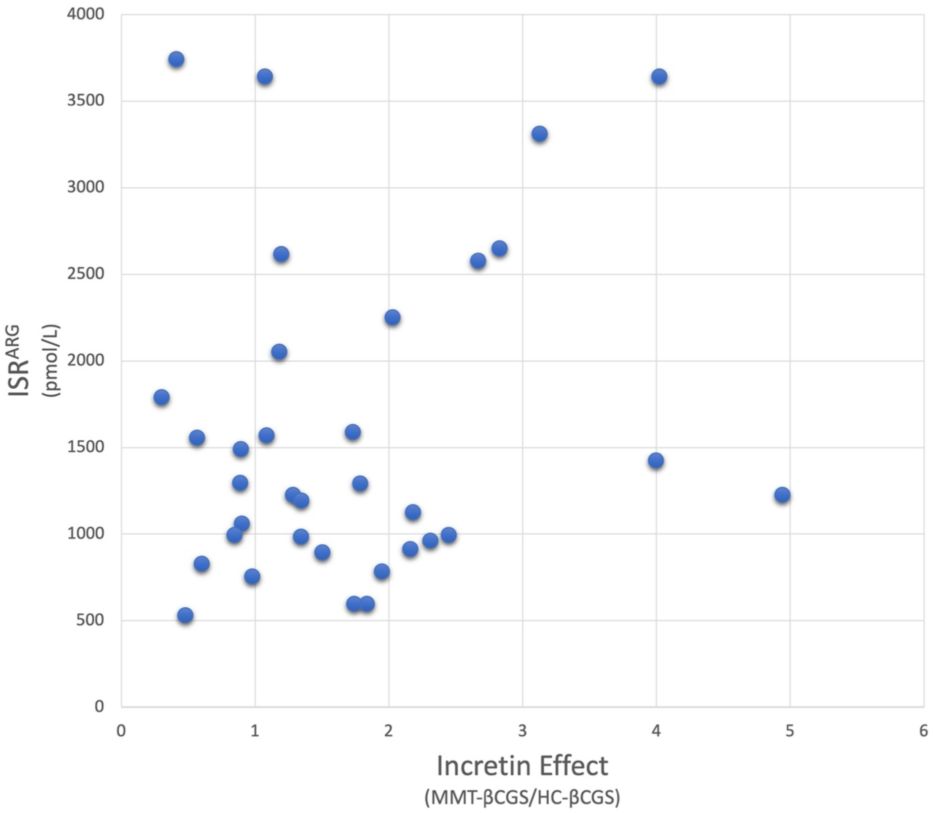

Supplemental Figure 1. Linear regression between ISR^{ARG}, and indirect index of total β-cell functional mass, and incretin effect (IE) values shows no correlation between β-cell functional mass and IE impairments in non-diabetic subjects.

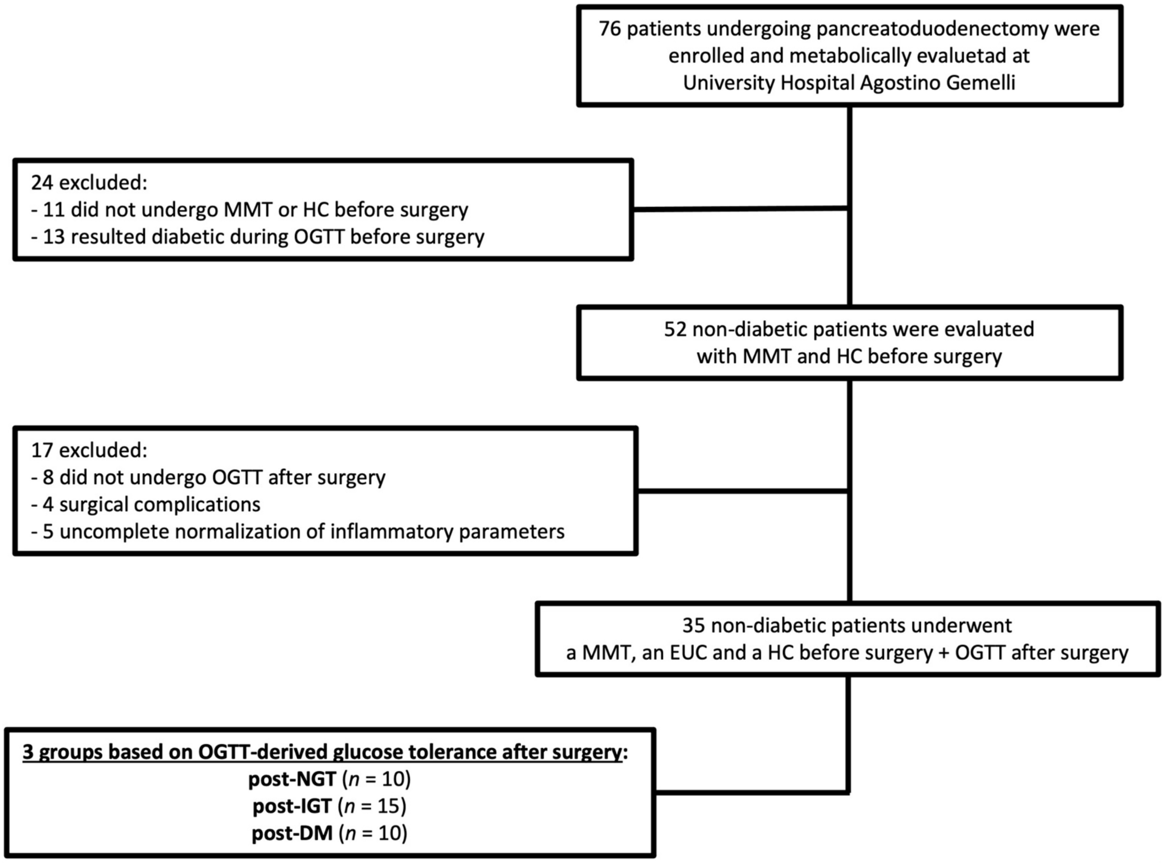

Supplemental Figure 2. Flow diagram of study participants
